# Assessing Torquetenovirus (TTV) as a Biomarker for Immune Responses to SARS-CoV-2 mRNA Vaccines in People Living with HIV and Healthy Individuals

**DOI:** 10.3390/vaccines13020153

**Published:** 2025-02-01

**Authors:** Claudia Minosse, Pietro Giorgio Spezia, Valentina Mazzotta, Giulia Matusali, Silvia Meschi, Francesca Colavita, Davide Mariotti, Stefania Notari, Alessandra Vergori, Daniele Focosi, Enrico Girardi, Andrea Antinori, Fabrizio Maggi

**Affiliations:** 1 Laboratory of Virology, National Institute for Infectious Diseases Lazzaro Spallanzani-IRCCS, 00149 Rome, Italy; 2 Clinical and Research Department, National Institute for Infectious Diseases Lazzaro Spallanzani-IRCCS, 00149 Rome, Italy; 3 Cellular Immunology and Pharmacology Laboratory, National Institute for Infectious Diseases Lazzaro Spallanzani-IRCCS, 00149 Rome, Italy; 4 North-Western Tuscany Blood Bank, Pisa University Hospital, 56124 Pisa, Italy; 5 Scientific Direction, National Institute for Infectious Diseases Lazzaro Spallanzani-IRCCS, 00149 Rome, Italy

**Keywords:** TTV, COVID-19, vaccination, anti-Spike antibodies, neutralizing antibodies, people living with HIV

## Abstract

**Background**: Torquetenovirus (TTV) viremia is increasingly recognized as a marker of immune competence. In the context of COVID-19, TTV viral load (VL) has been shown to predict anti-Spike antibody levels in severely immunocompromised patients. This study aimed to evaluate whether pre-vaccine TTV VL could predict humoral and cellular immune responses to SARS-CoV-2 mRNA vaccines in people living with HIV (PLWH) and healthy individuals (HP). **Methods**: TTV VL was measured via real-time PCR in serum samples collected before the second and third doses of mRNA vaccines in 93 PLWH and 48 HP (second dose) and 255 PLWH and 48 HP (third dose). Immune responses were assessed through anti-SARS-CoV-2 receptor-binding domain (RBD) IgG, neutralizing antibodies, and IFN-γ release. Statistical analyses included correlation studies between TTV VL and vaccine-induced immune responses. **Results**: TTV VL did not significantly correlate with anti-RBD IgG or neutralizing antibody levels in either cohort; highlighting its limited predictive value for humoral responses in relatively immunocompetent populations. However, a strong inverse correlation was observed between TTV VL and IFN-γ release after the third, but not the second, vaccine dose. These findings suggest that higher TTV VL, indicative of reduced immune competence, may impair T-cell-mediated immunity to vaccines. **Conclusions**: In virologically suppressed PLWH and HP, TTV VL is not a reliable predictor of humoral immune responses to COVID-19 vaccines. However, its inverse relationship with cellular responses warrants further investigation in more immunosuppressed populations. These results reinforce the continuum model of TTV VL as a biomarker, with predictive utility increasing alongside the degree of immunosuppression

## 1. Introduction

Torquetenovirus (TTV; from *torques* and *tenuis*, Latin for ‘necklace’ and ‘thin’, respectively) is the prototype of a large group of small DNA viruses with a circular, negative-sense, single-stranded genome. The TTV DNA molecule is about 3.8 kilobases in length and includes a coding region that covers more than 70% of its genome. This coding region is divided into at least four major, partially overlapping open reading frames (ORFs), while the remaining portion does not have any known coding functions (untranslated region, UTR) [1,2]. Sequence divergence varies across the genome. The UTR is highly conserved with over 90% sequence identity between isolates, and contains a GC-rich tract. Conversely, the translated region shows significant diversity, with ORF1 containing a hypervariable region. Since its discovery in 1997, numerous closely related TTV sequences have been identified, demonstrating a remarkable level of genetic variability in this virus. TTV is now classified into at least 20 major species, each of which consists of numerous genotypes, grouped into the *Alphatorquevirus* genus of the Anelloviridae family [3]. Historically, the virus has been classified as an orphan virus. However, recent research employing metagenomic techniques has identified TTV as the most prevalent constituent of the human blood virome [4,5]. Multiple TTV genotypes are sequentially acquired early in life throughout various routes, resulting in an extremely high global prevalence. TTV persists in T lymphocytes [6], with chronic and stable viremia in healthy people at approximately 2.0–3.0 Log copies/mL of blood [7]. Due to its widespread prevalence and close connection with the host’s immune system, TTV is being increasingly studied as a substitute marker for functional immune competence [8,9]. Elevated TTV DNA levels have been documented in cases of sepsis [10], HIV infection [11,12,13], newly diagnosed untreated solid tumors, autologous or allogeneic hematopoietic stem cell transplants [14,15], and solid organ transplants [16,17,18,19,20,21,22]. An important randomized controlled trial is currently evaluating the use of TTV to tailor maintenance immunosuppression in kidney transplant patients [23].

In the context of COVID-19, our research group [24,25] and others [26,27,28,29,30,31] have demonstrated that pre-vaccination TTV viremia can predict anti-Spike antibody levels post-vaccination in severely immunocompromised populations, such as solid organ transplant recipients. That is likely attributable to the fact that higher pre-vaccination viremias are correlated with poor immune competence, resulting in a lower immune response to the vaccine. Conversely, lower viremias are associated with a more active immune system, leading to a higher vaccine response. To date, no studies have investigated the relationship between TTV loads and COVID-19 vaccine responses in fully or relatively immunocompetent patients, with the hypothesis that its predictive value for humoral responses needs to be contextualized to immunosuppression levels.

Here, we further explored whether this predictive capability is applicable to people living with HIV (PLWH) and healthy individuals.

## 2. Materials and Methods

### 2.1. Study Design and Population

This study investigated immune responses to COVID-19 vaccination in two groups: people living with HIV (PLWH) as part of the HIV-VAC study and healthy individuals (HP) participating in the HCW-VAC study. The research was conducted at the National Institute for Infectious Diseases (INMI) Lazzaro Spallanzani in Rome. Participants were eligible if they had received either two or three doses of a wild-type SARS-CoV-2 mRNA vaccine (BNT162b2 or mRNA-1273) and had serum samples available from the first (T0) or third (T3) vaccine dose. To avoid confounding factors, participants with a documented history of SARS-CoV-2 infection were excluded from study population.

PLWH consistently attend our hospital’s HIV clinic and are receiving antiretroviral therapy. Antibody responses were assessed in 93 PLWH and 48 HP individuals after the second vaccine dose, and in 255 PLWH and 48 HP individuals after the third dose.

### 2.2. TTV DNA Detection and Quantification

Viral DNA was extracted from serum using the QIAsymphony platform (Qiagen, Hilden, Germany). TTV viral load (VL) in serum samples collected at T0 and T3 was quantified using the CE-IVD-certified TTV R-GENE^®^ kit (bioMérieux, Marcy-l’Etoile, France). Amplification was carried out on the Rotor-Gene Q2plex (Qiagen, Hilden, Germany) following the manufacturer’s protocol. The TTV R-GENE^®^ kit provides standards to create a quantification curve, with TTV VL expressed in log copies/mL. Each experiment included a negative control. A value of 1.0 log was assigned to undetectable TTV VL. Procedures for quantification, specificity assessment, sensitivity assessment and precision analysis have been described previously [32,33].

### 2.3. SARS-CoV-2 IgG Antibody Testing

The Abbott Architect^®^ SARS-CoV-2 IgG II Quant Assay (Abbott, North Chicago, IL, USA) was employed to measure anti-SARS-CoV-2 Spike receptor-binding domain (RBD) IgG antibodies in serum samples collected 2–4 weeks following the second or third vaccine dose. Antibody levels were converted to binding antibody units (BAU)/mL using a multiplication factor of 0.142, as per the World Health Organization SARS-CoV-2 immunoglobulin standard. The assay’s quantification range was 1.0 to 11,360 BAU/mL, with a positivity cutoff set at 7.1 BAU/mL. Participants with anti-RBD IgG levels ≥ 7.1 BAU/mL were classified as vaccine responders, while those with levels < 7.1 BAU/mL were considered non-responders.

### 2.4. SARS-CoV-2 Microneutralization Assay

The microneutralization assay (MNA) was carried out using the live SARS-CoV-2 Wuhan-D614G strain (GISAID accession ID EPI_ISL_568579). This procedure adhered to previously established protocols to assess the neutralizing capacity of the samples against the virus [24]. In summary, heat-inactivated serum samples were diluted and then incubated with 100 TCID_50_ of SARS-CoV-2 at 37 °C for 30 min. Next, 100 μL/well of the SARS-CoV-2/serum mixtures were added to 96-well tissue culture plates containing sub-confluent Vero E6 cell (ATCC Number CRL-1586) monolayers. After 48 h of incubation, the microplates were examined under a light microscope to detect any cytopathic effect (CPE). The serum dilution that inhibited at least 90% of the CPE was designated as the neutralization titer (MNA_90_). Values of 1:10 or higher were considered positive.

### 2.5. Interferon-Gamma ELISA Assay

The level of interferon-gamma (IFN-γ) was assessed using a previously established protocol [34]. Briefly, peripheral blood was collected in heparin tubes and either stimulated with a peptide pool covering the Spike protein (Prot-S code 130126701; Prot-S1 code 130127048; Prot-S+ code 130127312; Miltenyi Biotech, Bergisch Gladbach, Germany) or unstimulated. Stimulation was performed with 5% CO_2_ at 37 °C. Staphylococcal enterotoxin B (SEB) superantigen was used as a positive control and the spontaneous release of cytokines was measured in the unstimulated culture. Plasma samples were collected after 16−20 h of stimulation and stored at −80 °C. An automated ELISA system (ELLA™, Bio-Techne, Minneapolis, MN, USA) was used to measure IFN-γ levels in plasma samples. The detection limit of the assay was 0.17 pg/mL, and a positive IFN-γ response was defined as ≥12 pg/mL.

### 2.6. Statistical Analysis

Categorical data were expressed as frequencies and percentages, while continuous variables were summarized using means (standard deviation), medians, and interquartile ranges (25th and 75th percentiles), as appropriate. Fisher’s exact test and the Chi-square test were employed to compare categorical variables, whereas the Mann−Whitney U-test, a non-parametric method, was used for continuous variable comparisons. To assess potential linear relationships between two continuous variables, the Spearman correlation, also non-parametric, was applied. All statistical tests were two-tailed, with a significance threshold of *p* < 0.05. Analyses were performed using Prism v.8.0.2 software.

## 3. Results

### 3.1. Study Population

A total of 444 serum samples were collected: 348 from PLWH and 96 from HP groups. Specifically, 93 samples from PLWH were assessed after the second vaccine dose (recruitment period: 24 March 2021–21 April 2021), and 255 after the third vaccine dose (recruitment period: 20 September 2021–1 February 2022). From the HP group, 48 samples were assessed after the second vaccine dose (recruitment period: 4 January 2021–8 January 2021), and 48 after the third vaccine dose (recruitment period: 25 October 2021–4 December 2021). None of the participants had a prior SARS-CoV-2 infection, as confirmed by serological testing for anti-SARS-CoV-2 Nucleoprotein IgG before T0 and at T3, as well as anti-SARS-CoV-2 Spike-RBD IgG levels before T0. The demographic and laboratory characteristics of the study population are presented in Table 1 and Table 2.

### 3.2. TTV VL and Anti-SARS-CoV-2 RBD IgG Response

**Figure 1:**
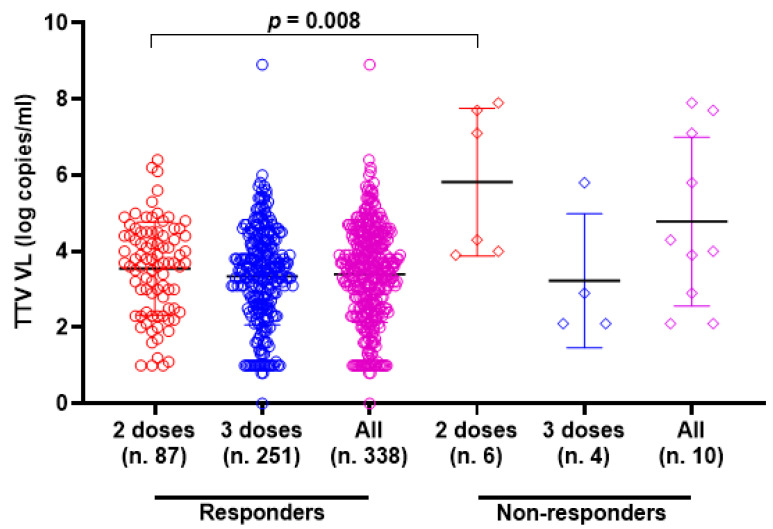
Pre-vaccine TTV VL and anti-SARS-CoV-2 IgG post-vaccination response in PLWH (circle and square represented the responders and non-responders, respectively) after the second dose (red), third dose (blue) and overall (purple). Median, upper, and lower quartiles are indicated by horizontal lines.

**Figure 2:**
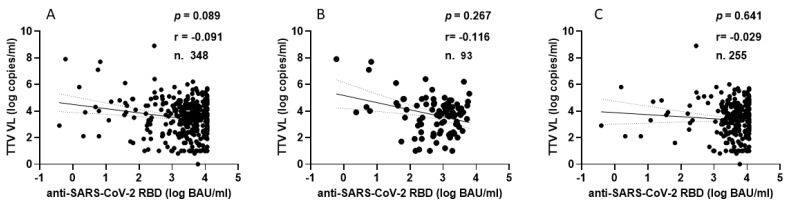
Correlation between pre-vaccine TTV VL and post-vaccination anti-SARS-CoV-2 antibody levels in PLWH: overall (**A**), after second dose (**B**), and after third dose (**C**).

TTV VL was assessed in serum samples collected at T0 and T3 from both PLWH and HP. As detailed in Table 1, 330 of the 348 serum samples (94.8%) from PLWH were positive for TTV DNA, with a median viral load of 3.6 Log copies/mL. There was no significant difference in TTV prevalence between T0 and T3, but a moderately significant difference was observed between the median TTV VL (*p* = 0.047). In Table 2, 59 out of 96 serum samples (61.5%) tested positive for TTV DNA, with a median VL of 2.5 Log copies/mL. No significant difference was found in the prevalence of TTV between T0 and T3 or between the VL medians. Table 3 presents patient characteristics and clinical parameters in PLWH, categorized by anti-SARS-CoV-2 seroconversion after receiving two or three vaccine doses. Before the initial two-dose vaccination (T0), the TTV VL was observed to be 2.0 Log higher in individuals who did not respond to the vaccine compared to those who did (3.7 vs. 5.7 Log copies/mL, *p* = 0.008). However, this significant relationship between pre-vaccination TTV VL and the magnitude of the anti-RBD IgG response was not detected after the third vaccine dose was administered. Figure 1 graphically presents the pre-vaccination TTV VL values in relation to the anti-SARS-CoV-2 IgG response grouped by doses of the vaccine. The correlation between TTV VL and serum anti-RBD IgG levels was examined. As shown in Figure 2, no significant inverse correlation was observed between pre-vaccination TTV VL and anti-SARS-CoV-2 RBD IgG levels. The absence of correlation was evident in all samples (r = −0.091; *p* = 0.089; Figure 2A) and remained consistent within subgroups based on the number of vaccine doses administered: after two doses (r = −0.116; *p* = 0.267) and after three doses (r = −0.029; *p* = 0.641), as illustrated in Figure 2B,C.

**Figure 3:**
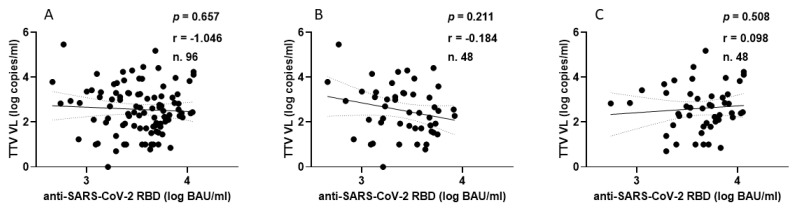
Correlation between pre-vaccine TTV VL and post-vaccination anti-SARS-CoV-2 antibody levels in HP: overall (**A**), after second dose (**B**), and after third dose (**C**).

In the HP group, every individual showed anti-SARS-CoV-2 seroconversion after receiving two or three doses of vaccine. Figure 3 shows the correlation between TTV VL and serum levels of anti-RBD IgG. In this population group, no significant correlation was observed between pre-vaccination TTV VL and serum anti-SARS-CoV-2 RBD IgG levels.

### 3.3. TTV VL and Anti-SARS-CoV-2 Neutralizing Antibodies Response

**Figure 4:**
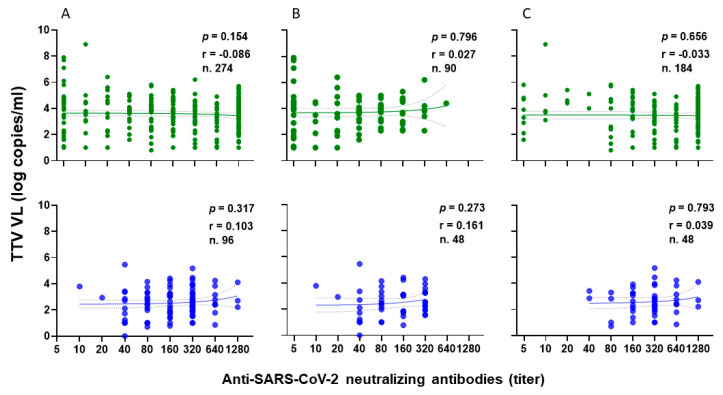
Correlation between pre-vaccine TTV VL and post-vaccination anti-SARS-CoV-2 neutralizing antibody titers in PLWH (green) and in HP (blue): overall (**A**), after second dose (**B**), and after third dose (**C**).

The correlation between TTV VL and levels of anti-SARS-CoV-2 neutralizing antibodies (nAbs) was examined in 274 out of 349 serum samples of PLWH following the administration of the second and third vaccine doses. In total, nAbs were present in 244 out of 274 (89.0%) samples (Figure 4A, green). Specifically, nAbs were detected in 69 out of 90 (76.7%) samples after the second vaccine dose (Figure 4B, green) and in 175 out of 184 (95.1%) samples after the third vaccine dose (Figure 4C, green). Out of 244 samples, 171 (36.2%) revealed nAbs titers ≥ 1:160 (18 and 153 samples after the second and third vaccine doses, respectively). There is no statistical difference between pre-dose TTV VL in PLWHs with nAbs responses and those with no nAbs responses (3.6 versus 3.9 Log copies/mL, respectively). Furthermore, there was no correlation observed between pre-vaccine TTV VL and the levels of nAbs generated following the administration of the second and third vaccine doses, as illustrated in PLWHs (Figure 4, green).

For the HP group, the correlation between TTV VL and nAbs levels was examined after the second and third vaccine doses in all 96 serum samples. nAbs were present in 100% of samples. Specifically, 67 of 96 samples (69.8%) had nAbs titers ≥ 1:160 (Figure 4A, blue). In particular, 25 and 42 samples had nAbs titers ≥ 1:160 after the second (Figure 4B, blue) and third vaccine doses (Figure 4C, blue), respectively. No correlation was found between TTV VL and nAbs levels after either vaccine dose, as shown in (Figure 4A–C, blue).

### 3.4. TTV VL and Cell-Mediated Immune Response

In a subset of 90 and 153 PLWH and in 48 and 41 HP after the second or third doses of the SARS-CoV-2 vaccine, specific T-cell responses to S-peptides were evaluated.

**Figure 5:**
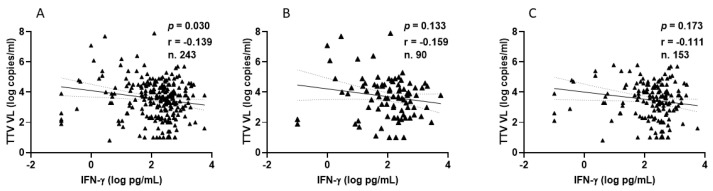
Correlation between pre-vaccine TTV VL and post-vaccination IFN-γ levels in PLWH: overall (**A**), after second dose (**B**), and after third dose (**C**).

Among PLWHs, 77 out of 90 (85.6%) showed a positive IFN-γ response after the second vaccination, with a median level of 210.9 pg/mL (IQR 80.3–436.9). Similarly, after the third vaccine dose, 137 out of 153 patients (89.5%) showed a positive response, with a median IFN-γ level of 283.8 pg/mL (IQR 124.2–533.1). Figure 5 shows the significant inverse correlations observed between pre-vaccine TTV VL and IFN-γ levels measured overall, after the second and after third doses of vaccine. This significant correlation is not evident when the values are considered separately (Figure 5B,C).

**Figure 6:**
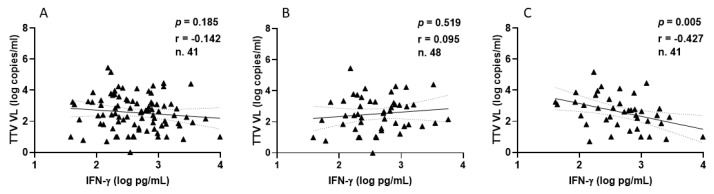
Correlation between pre-vaccine TTV VL and post-vaccination IFN-γ levels in HP: overall (**A**), after second dose (**B**), and after third dose (**C**).

Focusing on HPs, all samples demonstrated a positive IFN-γ response, with a median level of 393.8 pg/mL (IQR 194.5–812.5) following the second vaccination. After the third vaccine dose, 41 out of 48 HPs (85.4%) exhibited a positive IFN-γ response, with a median level of 448.9 pg/mL (IQR 188.6–995.3). The correlations between TTV VL and IFN-γ levels measured overall (Figure 6A), after the second (Figure 6B), and third (Figure 6C) vaccine doses are depicted in Figure 6. A negative correlation was observed between pre-vaccine TTV VL and IFN-γ levels measured post-third dose. This significant correlation was not observed when considering values post-second dose or overall (Figure 6A,B).

## 4. Discussion

While previous studies have demonstrated the utility of TTV VL in predicting immune responses in severely immunocompromised individuals, such as solid organ transplant recipients [26,27,28,29,30,31,35], its applicability in relatively immunocompetent populations remained uncertain.

Our results reveal no significant inverse correlation between pre-vaccination TTV VL and anti-SARS-CoV-2 RBD antibody levels or nAbs titers after the second or third dose in either PLWH or HP. This finding differs from data on severely immunocompromised individuals, where higher TTV VL correlates with weaker vaccine antibody responses. Among PLWH, we observed that non-responders to the second vaccine dose exhibited significantly higher pre-vaccination TTV VL compared to responders. However, this association disappeared after the third dose, suggesting that booster doses may help equalize antibody responses, even among individuals with initially higher TTV VL. These findings suggest that TTV VL is not a reliable biomarker for predicting antibody responses to COVID-19 vaccines in relatively immunocompetent individuals. In contrast, a significant inverse correlation was observed between pre-vaccination TTV VL and IFN-γ levels following T-cell stimulation with the Spike protein, particularly after the third vaccine dose. This trend was evident in both PLWH and HP. These results suggest that higher TTV VL, indicative of greater immune impairment, may limit the ability to mount a robust T-cell-mediated immune response to vaccination. While this highlights a potential role for TTV VL as an indicator of cellular immune competence, its predictive value remains limited in cohorts with relatively intact immune systems. Interestingly, virologically suppressed PLWH displayed immune responses comparable to those of HP, both in terms of antibody production and T-cell-mediated immunity.

The study has several strengths that enhance its significance and reliability. First, by excluding participants with a history of SARS-CoV-2 infection, it minimizes confounding factors related to prior infection-elicited immunity, ensuring a clearer evaluation of vaccine-induced responses. Also, it assesses a wide range of immune responses to mRNA COVID-19 vaccines, including humoral responses and cell-mediated immunity. This multifaceted approach better explains the relationship between TTV VL and vaccine-induced immunity. The research also includes both PLWH and HP, allowing for a comparison of immune responses across populations with varying degrees of immune competence, which strengthens the generalizability of the findings. By studying virologically suppressed PLWH, the study highlights the immune competence of this population, reinforcing their comparability to healthy individuals in terms of vaccine responsiveness. This finding has implications for public health strategies targeting PLWH.

The study also has some limitations. While the overall sample size was substantial, certain subgroup analyses, such as those involving non-responders among PLWH, could benefit from larger numbers to enhance statistical power. Again, the study primarily focused on short-term immune responses to vaccination. Longitudinal studies assessing the role of TTV VL in predicting long-term vaccine efficacy and durability of immune responses would provide a more comprehensive understanding of potential TTV applications.

In conclusion, our research demonstrates that TTV VL is not an effective predictor of antibody responses to COVID-19 vaccines in fully and relatively immunocompetent individuals. However, the correlation with immune responses indicates that TTV VL could be useful as an indicator of immune system health in specific situations, especially when used along with other potential biomarkers, such as CD4 count [36]. The predictive value of TTV VL appears to follow a continuum based on the degree of immunosuppression, with greater utility observed in more severely immunosuppressed populations.

Looking ahead, integrating TTV VL with other biomarkers may improve its predictive accuracy. Establishing standardized clinical definitions of immunosuppression would also facilitate the broader application of markers like TTV VL. While TTV VL offers insights into immune status, its utility as a biomarker should remain focused on populations with significant immunosuppression, where it may provide the most meaningful clinical value.

## 5. Conclusions

This study highlights the predictive value of TTV VL for immune responses in relatively healthy cohorts, showing its utility in assessing cellular immune competence versus humoral responses. The findings support the hypothesis that contextualizing TTV VL’s applications based on immunosuppression levels is important, demonstrating its relevance in clinical and immunological contexts.

## Figures and Tables

**Table 1:** PLWH characteristics, grouped by vaccine dose.

Parameter	PLWH	
	First + Second Dose (n. 93)	Third Dose (n. 255)
Age in years–median (IQR)	56 (48–61)	55 (48–60)
Male–n. (%)	74 (79.6)	200 (78.4)
CD4 ^a^–median cells/mm^3^ (IQR)	409 (194–572)	397 (231–639)
CD4/CD8 ^a^–median cells/mm^3^ (IQR)	0.5 (0.2–0.9)	0.5 (0.3–0.9)
Pre-dose TTV DNA positive–n. (%)	90 (96.8)	240 (94.1)
Pre-vaccine Log copies/mL TTV VL–median (IQR)	3.8 (2.6–4.5)	3.4 (2.5–4.2) ^b^
Pre-vaccine Log copies/mL TTV VL in CD4 < 200–median (IQR)	4.6 (2.8–5.3)	4.3 (3.1–5.4)
Pre-vaccine Log copies/mL TTV VL in CD4 > 200–median (IQR)	3.7 (2.9–4.3)	3.5 (2.6–4.1)
Anti-SARS-CoV-2 RBD median Log BAU/mL (IQR)	3.0 (2.4–3.3)	3.7 (3.4–4.0)

^a^ CD4 and CD4/CD8 values were available for 91/93 PLWH with response anti-SARS-CoV-2 RBD evaluated after the 2nd vaccine dose and 198/255 with response anti-SARS-CoV-2 RBD evaluated after the 3rd vaccine dose. ^b^ Statistically different from the median TTV VL in PLWH with one plus two doses of the vaccine (*p* = 0.047). Abbreviations: PLWH, people living with HIV; IQR, interquartile range; TTV VL, TTV viral load.

**Table 2:** HP characteristics, grouped by vaccine dose.

Parameter	HP	
	First + Second Dose (n. 48)	Third Dose (n. 48)
Age in years–median (IQR)	45 (32–52)	45 (32–52)
Male–n. (%)	15 (31.2)	15 (31.2)
Pre-vaccine TTV DNA positive–n. (%)	30 (62.5)	29 (60.4)
Pre-vaccine Log copies/mL TTV VL–median (IQR)	2.5 (1.7–3.3)	2.6 (2.0–3.2)
Anti-SARS-CoV-2 RBD median Log BAU/mL (IQR)	3.4 (3.2–3.7)	3.7 (3.4–3.8)

Abbreviations: HP, healthy people; IQR, interquartile range; TTV VL, TTV viral load.

**Table 3:** PLWH characteristics, grouped by response to anti-SARS-CoV-2 doses.

	Two Vaccine Doses			Three Vaccine Doses		
Parameter	R ^a^ (n. 87, 93.5%)	NR (n. 6, 6.5%)	*p*	R (n. 251, 98.4%)	NR (n. 4, 1.6%)	*p*
Age in years–median (IQR)	56 (48–60)	62 (50–67)	NS	55 (48–60)	63 (58–68)	0.029
Male–n. (%)	70 (80.5)	4 (66.7)	NS	197 (78.5)	3 (75.0)	NS
CD4–median cells/mm^3^ (IQR) ^b^	446 (217–575)	90 (55–298)	0.005	408 (241–644)	59 (29–74)	<0.001
CD4/CD8–median cells/mm^3^ ^b^ (IQR)	0.5 (0.3–0.9)	0.1 (0.1–0.2)	<0.001	0.5 (0.3–0.9)	0.1 (0.1–0.2)	0.002
CD4 < 200–n. (%) ^a^	19 (22.4)	4 (66.7)	0.015	35 (18.0)	4 (100.0)	<0.001
Pre-vaccine TTV DNA positive–n. (%)	84 (96.6)	6 (100.0)	NS	236 (94.0)	4 (100.0)	NS
Pre-vaccine Log copies/mL TTV VL–median (IQR)	3.7 (2.5–4.4)	5.7 (4.0–7.8)	0.007	3.4 (2.5–4.2)	2.5 (2.1–5.1)	NS

^a^ Responders (R) were defined as patients with anti-SARS-CoV-2 RBD titers of 7.1 BAU/mL or higher. Non-responders (NR) were defined as patients with anti-SARS-CoV-2 RBD titers less than 7.1 BAU/mL. ^b^ CD4 and CD4/CD8 values were available for 91 PLWH after two vaccine doses (85 responders, six non-responders) and for 198 after three vaccine doses (194 responders, four non-responders). Abbreviations: IQR, interquartile range; NS, not significant; TTV VL, TTV viral load.

## Data Availability

The data that support the findings of this study are available from the corresponding author upon reasonable request.
